# Discussions on target theory: past and present

**DOI:** 10.1093/jrr/rrt075

**Published:** 2013-06-03

**Authors:** Takuma Nomiya

**Affiliations:** National Institute of Radiological Sciences, 4-9-1, Anagawa, Inage-ku, Chiba, 263-8555, Japan

Target theory is one of the essential concepts for understanding radiation biology. Although many complex interpretations of target theory have been developed, its fundamental principle is that ‘inactivation of the target(s) inside an organism by radiation results in the organism's death’. The number of ‘targets’ and the locations of these ‘targets’ inside an organism are not always clear, although the ‘target’ is considered as a unit of biological function. Assuming that when an average one-hit dose (inactivation of one target) per organism is used and one-hit of irradiation results in the organism's death, then the probability of survival [*P*(1) = *S*] is expressed by:
(1)
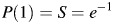



When the number of organism units and the number of trials are sufficiently large, the survival rate of an organism (cells, bacteria, etc.) is about 37% (*e*^*−1*^). When a dose that causes an average of *x* hits inside an organism unit is used for irradiation, the survival rate of a cell population (*P*(*x*) = *S*) is expressed by:
(2)
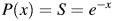



Equation (2) was described in a paper published in 1962 [1]. Before this paper was published, Lea *et al*. had reported the results of their detailed studies on the effects of radiation on bacteria and viruses [2–4]. The results of their experiments suggested that the survival probability of irradiated organisms decreased exponentially with increased irradiation dose. Their results corresponded well with the calculated survival rates given in equation (2).

With regard to a multitarget model, the survival probability of a cell is represented well by:
(3)
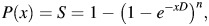



where *S* = probability of survival, *D* = a dose that causes a mean of one-hit per cell (mean lethal dose), *x* = number of hits per cell, *n* = number of targets (required number of hits for cell death).

**Fig. 1.**
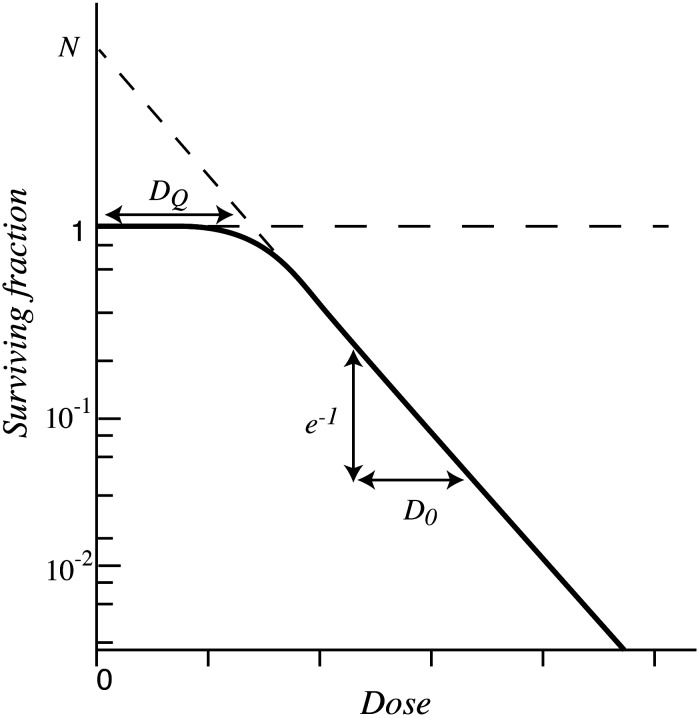
Cell-survival curve of multitarget model in conventional target theory.

This equation, which was used in the review by Little *et al*. in 1968, has now been established as a multihit target theory model [5]. Figure 1 shows equation (3), and this graph is introduced in the chapter of basic radiation biology in almost all textbooks of radiation oncology. This graph represents the cell-survival curve and the radiosensitivity of the cell line using ‘*D*_*Q*_ (quasi-threshold dose),’ ‘*D0* (slope),’ and ‘*N* (extrapolation number)’.

Considering the origin of this equation, a similar equation could be found in a previous article on radiation effects [6]:
(4)
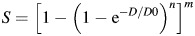

where *S* = probability of survival, *D0* = a dose that causes a mean of one-hit per cell (mean lethal dose), *D* = irradiated dose, *n* = heteroploid number (1: haploid, 2: diploid, 3: triploid), *m* = number of targets (required number of hits for cell death).

They derived the following equation for *n* = 2 and *m* = 1:
(5)
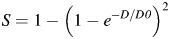



Equations (4) and (5) are quite similar. However, it appears that there are differences in the position of the multiplier and the origin of the expression that represents the number of targets.

Two papers have been cited that led to equation (5) [7, 8]. Luria *et al*. attempted to formulate a mathematical model of phage inactivation by radiation on the basis of their experimental results [7]. They considered the following model: one phage consists of ‘*n*’ independent units (loci). A dose that causes γ hits per unit is used for irradiation, and one or more hits cause inactivation of a unit (locus). Infection of ‘*k*’ phages in a single bacterium yields new phages. At least one specific locus (e.g. locus A) of these ‘*k*’ phages survives, which makes this locus ‘active’. A new phage arises only when all ‘*k*’ loci are active, otherwise no new phages arise.

The probability that an active phage (y_*k*_) will be produced is expressed by:
(6)
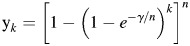



Although a model of a cell that had ‘*n*’ targets directly applied equation (6) in one paper [6], it would seem that verification of this application is required.

In another paper [8], as in similar papers, a survival probability using a one-hit target model was expressed by:
(7)
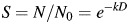



where *N* = cell population after irradiation, *N*_*0*_ = cell population before irradiation, *D* = irradiated dose, *k* = constant.

The survival probability of a multihit target model in this paper [8] was expressed as follows:
(8)
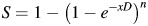



Equation (8) is similar to the aforementioned equations. According to the authors' definition: ‘The assumption here is not simply that *n* hits per organism are required, but that each of *n* particles within the organism must be hit at least once.’ In their model, a cell is not inactivated by simply being hit by *n* particles. This concept is based on the ‘multitarget single-hit’ model that was proposed later [9]. This model defines that there are more targets than just one in an organism, and inactivation of all the targets leads to death of the organism.

On the other hand, it is well known that the distribution of a cell population based on the number of hits conforms to a Poisson distribution, and descriptions of Poisson distributions are also found in some previously cited papers [2, 4, 6, 8]. The Poisson distribution describes the probability that a phenomenon that occurs ‘*n*’ times on average will occur exactly ‘*k*’ times. This probability is defined by:
(9)
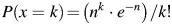



In the one-target model (*n* = 1), the surviving cell population corresponds well with the Poisson distribution; thus, there is continuity between this model and the Poisson distribution.

Because each hit is not distinguished in the Poisson distribution, it can be said that the probability of occurring ‘*k*’ times in the Poisson distribution is the same as the probability of ‘*k*-hits’ in the ‘single-target multihit’ model [9]. For example, inactivation of all three targets lead to death of an organism in the ‘3-target 1-hit’ model (an organism does not die even if more than three hits occurred in some cases), whereas an organism will surely die with three hits in the ‘1-target 3-hit’ model. Although there are several papers that have indiscriminately described the above two models, these models should be discussed with a clear distinction. Further, verification about that ‘multitarget single-hit’ model (shown by equation (3) or equation (8)) is described as the *de facto* standard model of target theory.

Aside from the question of whether the ‘multitarget single-hit’ model most appropriately shows the real phenomenon or not, the remaining ‘multitarget single-hit’ model shows characteristics of cell lines. This model seems to successfully represent the characteristics of cell-survival curves by using simple factors such as ‘*D*_*Q*_’, ‘*D*_*0*_’ and ‘*N*’, and it is easy to understand the meaning of each factor in this model. However, dissociation from actual cell-survival curves has been pointed out, particularly in cell-survival curves with high ‘*D*_*Q*_’ or high extrapolation numbers [10]. Although this model is mentioned in many textbooks of radiation biology, it is rarely used in present clinical and basic studies, and the Linear-Quadratic (LQ) model has been used for target theory after that [11].

The target theory is consistent with measured survival curves in the case of the 1-hit model, e.g. for certain bacteria, and is also consistent with Poisson distribution (probability of 1-hit). But the dissociation becomes larger when it comes to the ‘multitarget’ model. This is thought to be the reason why target theory is rarely used. But the LQ model has not been able to reproduce the cell-survival curves completely, so appropriate modification of the target model will make it more usable than any other models used at present.
